# Urban Households’ Biomass Energy Fuel Stacking and Its Implications for Sustainable Development Goal Seven: The Case of Jimma Town, Ethiopia

**DOI:** 10.1002/gch2.202100154

**Published:** 2022-02-18

**Authors:** Gudina Terefe Tucho, Mulunesh Deti, Dessalegni Dadi, Tizita Teshome

**Affiliations:** ^1^ Department of Environmental Health Sciences and Technology Jimma University Jimma Ethiopia

**Keywords:** biomass energy, cooking energy, energy profiles, Ethiopia, Jimma, SDG7, urban households

## Abstract

Sustainable development goal seven aims to provide access to affordable, reliable, sustainable, and modern energy for all by 2030, but its progress and energy stacking conditions have not been evaluated. This study aims to assess the urban household energy profile and link its state to sustainable development goals. This study employs a cross‐sectional study design on 265 households selected by systematic random sampling from a town called Jimma in Ethiopia and collects the data using interview‐based semi‐structured questionnaires. The study obtains information from all the selected households. The results show that more than 80% of the households have a grid electricity connection, but more than 85% of the households regularly use firewood and charcoal for cooking. On average, households use about 1236 kg of firewood and 630 kg of charcoal per year. Most households report power interruption, inability to afford electricity costs, and personal preferences for relying on biomass energy for cooking. Over 98% of the households use electricity for lighting, but few use it for appliances. This shows an inherent challenge attributed to the cooking energy services provided by biomass energy sources despite the accessibility of electricity. This shows the significant impacts of biomass energy stacking which connection to electricity alone cannot solve.

## Graphical abstract

Sustainable development goal seven aims to achieve access to affordable, reliable, sustainable, and modern energy for all by 2030. However, the finding from this study indicates that urban households will continue using biomass energy and it is less likely to achieve the goal without enabling policy focusing on local conditions.

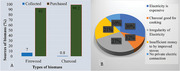

## 1 Background

Having access to modern energy services is essential for societal well‐being and livelihood change. A modern energy service for countries in tropical regions typically includes having reliable and affordable access to an improved cooking facility and connecting to electricity.^[^ ^1^, ^2^ ^]^ According to the International Energy Agency (IEA) report, by 2030, the number of people remaining without access to improved cooking energy technology will increase to over 2.5 billion due to the increasing population in developing countries. In sub‐Saharan Africa, Nigeria, Ethiopia, the Democratic Republic of Congo, Tanzania, and Kenya are countries where most people do not have access to a clean cooking facility. In principle, the cooking demand variation exists between developing and developed countries and between urban and rural due to varying socioeconomic and technological conditions. In western and temperate climates, the household energy demand's main share is for heating and appliances.^[^ ^3^ ^]^ On the one hand, the difference between rural and urban households’ energy demand is problematic because of the large majority of the poor urban households’ inability to afford modern cooking technologies.

Urban households are potentially accessible to grid connection and use for cooking, but charcoal and firewood remain primary energy sources for regular cooking.^[^ ^4^ ^]^ Poor urban households unable to afford electricity and its facilities remain reliant on biomass energy for all cooking purposes. Moreover, urban households are potentially accessible to semi‐processed food requiring less energy compared to unprocessed food. Shifting urban households to modern energy technology depends on the household's income,^[^ ^5^ ^]^ daily practice, and local conditions.^[^ ^6^ ^]^ Several studies have shown the effect of local energy use conditions, cooking behavior, and customs on adopting and sustaining improved energy technologies.^[^ ^7^, ^8^ ^]^ Households may not accept energy technologies unfit for local cooking habits and foods. Having a higher income alone is not sufficient to influence households to use electricity for cooking. Tanzania's study shows that households with higher incomes adopt modern fuels for lighting but remain on biomass for cooking.^[^ ^9^ ^]^

The steady growth in modern energy transition for the last 200 years has been linked to increasing levels of prosperity and economic opportunities across the world.^[^ ^10^ ^]^ Access to modern energy requires the provision and ability to afford and use modern and clean fuels for basic services. Access to modern energy technology to the poor in Africa is problematic, mostly due to traditional and modern energy systems and practices’ coexistence. The overlapping of modern and traditional energy use is also high in urban areas where households are potentially accessible to electrical energy. The simultaneous use of biomass fuels, kerosene, or electricity is a regular trend among economically better‐off families. The condition is more challenging when the sociocultural dimensions across sub‐Saharan Africa are taken into account. The energy transition is a slow process taking over 100 years.^[^ ^11^ ^]^ Major progress made in the twentieth century has been driven by consistent invention and technological changes.^[^ ^12^ ^]^ Access to mobile phone technology could be a good example.^[^ ^13^ ^]^ An increase in education and technology penetration would positively impact the transition.^[^ ^14^ ^]^ However, the transition in cooking energy technology may not be straightforward due to complex cooking behaviors and low socioeconomic conditions of the households to afford modern energy technologies.^[^ ^15^ ^]^ These conditions have been well illustrated with energy ladder model and fuel stacking conditions that linear transition cannot be achieved. Understanding household fuel choice and fuel switching characteristics is vital in search for policies to support a sustainable transition process.^[^ ^16^ ^]^

Most of the studies conducted and formulated policies mainly emphasized the rural energy transition dominated by biomass energy.^[^ ^17^, ^18^, ^19^ ^]^ Attention for rural energy transition originates from the assumption that urban households are connected to electricity and access all household services. However, these assumptions may not be realistic that urban households having access to electricity use it for cooing services, but this can be changed through time.^[^ ^20^ ^]^ It is believed that the energy transition in developing country is affected by energy stacking. However, the effects of the stacking could be variable as a trend study in India indicates, that fuel stacking decreased in lighting as electricity substitutes kerosene, but increased in cooking, as LPG unable to replace traditional biomass.^[^ ^21^ ^]^ Nevertheless, fuel stacking can be further complicated by socioeconomic, socioecological and cultural factors, level of access to biomass fuel and climate conditions and cooking technologies.^[^ ^22^ ^]^ Nevertheless, these factors are liable to change because of sociotechnical changes occurring as results of education and accessibility to energy technologies demanding electricity and local conditions. This can be understood from the energy use pattern of urban households potentially accessible to grid electricity connection.

Moreover, Sustainable Development Goal 7(SDG7) has been declared to ensure access to affordable, reliable, sustainable, and modern energy for all regardless of the country's geographic and socioeconomic conditions.^[^ ^23^ ^]^ The goal was declared five years ago, and ten years is remaining to achieve. Access to electric energy is deemed to provide clean and modern energy services for all households. Assessing the urban household electrical energy consumption, particularly for cooking, offers a full picture of the progress and possibility to accomplish within the remaining time. This means that the household energy profile would be expected to be dominated by electricity as the primary supplier. Achievement of the goal will be realized when the household energy transition become free of biomass stacking. However, most of the study results on fuel stacking in developing countries are too old and less likely applicable to indicate the progress to achieve sustainable development goal related to energy. The speed of diffusion with a digital mobile phone technology could be a good example^[^ ^24^, ^25^ ^]^ that recent information is needed to get a full picture of the progress of transition.

Ethiopia is among African countries with low level of electric energy access but moving with remarkable progress of renewable energy development with hydro and wind energy resources to provide clean energy to most of its people accessible to grid connection.^[^ ^4^ ^]^ Most of the urban households are potentially accessible to electricity connection and to use for all household energy services. However, information on urban household energy use pattern is not available to indicate the condition of fuel stacking which can be used to indicative of progress for the SDG 7. Understanding the progress in energy transition is vital for other sustainable development goals directly related to energy, such as access to education, access to health, poverty reduction and achieving women equality. Jimma town is among the Ethiopian towns and cities highly accessible to electricity connection because of its locational advantage as major electricity producing areas. The town is also among towns with increasing deficiency biomass fuel due to growing deforestation and strict regulation towards natural resources conservation. Most of the cities locating in the western, southern and south western regions of the country share more or less similar resources and electricity accessibility conditions, where the results from Jimma can be inferred. Despite accessibility to electricity, the extent to which electricity used for cooking in the town and its determinant factors is not yet well understood.

This study aims to assess the household energy profile of Jimma town and its implication in achieving related Sustainable Development Goals set for 2030. The pattern of household energy use is a clear manifestation of the expected transition towards the goal. The results from this study can be used as a reference to other towns and cities in the country sharing similar socioeconomic characteristics and resources availabilities. The finding is vital to re‐formulate energy supply strategies of the country as well as its Sustainable Development Goal 7 archiving strategies.

## 2 Methodology

### 2.1 Description of the Study Area and Its Population

The study was conducted in Jimma town, located in the Oromia region at 352 km from Addis Ababa, Ethiopia. The town is situated at geographical coordinates of 7°41’ N latitude and 36° 50’ E longitude. The town is found at an average altitude of about 1780 m above sea level and in the climatic zone locally known as “Woyna Daga” (1500–2400 m above sea level), which is ideal for human settlement. The town is generally characterized by a warm climate with an average high annual temperature of 26 °C and an average annual low temperature of 13 °C. The maximum precipitation occurs from June to August, with an average monthly rainfall of 240 to 275 mm, and the minimum rainfall occurs from December to February with an average of 38 mm. The limited temperature fluctuation is expected to show small contributions of energy consumption for space heating and cooling. According to the Central Statistical Agency 2007 report, this town has a projected total population of 120 960, of which 60 824 are males and 60 136 are females in 32 191 households or 128 306 populations according to the projection of world barometer.^[^ ^26^ ^]^

A community‐based cross‐sectional study was conducted on selected households of the town from April 15 to 25, 2019. The sample size was determined based on a single population proportion formula with a 95% confidence interval, margin of error of 5%,^[^ ^27^ ^]^ and a population proportion of 20%, which is a prevalence of the urban households having access to modern energy services.^[^ ^28^, ^29^ ^]^ Accordingly, 265 households were selected for the study by adding a 10% nonresponse rate for compensation.

The study was conducted by dividing the town into inner and outer kebeles (i.e., smallest administrative units) by considering their adjacency to rural areas, accessibility to the common forest for firewood collection, and population density. Accordingly, four kebeles (two from each) were purposely selected by proportionally allocating the sample size according to their total households. The number of households selected from four kebeles was determined by dividing the sample size by the number of households in each kebele. This approach was used to get a representative sample of the households of the town. Following that, a systematic random sampling technique was employed to select the households. The study considered households living for more than six months in the area. Households who were not willing to participate or with a mental health problem were excluded from responding to the questions, and thus the next households were considered instead. The study households were contacted after getting an ethical approval letter from Jimma university institute of health Institutional Review Board (IRB) and obtaining written consent from the respondents. The study considered women above the age of 18 years to respond to the questions because of the cultural burden of shouldering energy issues.

### 2.2 Data Collection and Analysis

The data were collected by using pretested interview‐guided semi‐structured questionnaires. The questionnaire was developed first in English and then translated into Afaan Oromo and Amharic and back‐translated to English with independent translators to check the questionnaires’ consistency and understandability. The questionnaire consisted of socioeconomic variables and questions related to household energy use patterns. The household energy consumption survey requested respondents to answer household energy sources, energy appliances, energy services, and reasons to use specific energy carriers and appliances for certain energy services. The questionnaires were pre‐tested on 5% of the population in other kebeles as a pilot study to check the questions’ ambiguity and consistency. Respondents were interviewed at their homes by trained data collectors. Prior to that, data collectors and supervisors were trained on data collection tools and how to obtain consent from respondents.

Moreover, the data quality was assured by assigning well‐qualified data collectors and site supervisors. Trained supervisors were assigned to correct any encountered problems during the field survey. All completed questionnaires were examined every day for their completeness and consistency by investigators and supervisors’ teams.

The data were entered into EPI‐data version 3.1 and exported into a statistical software package for social sciences (SPSS V.22) for analysis. First, a descriptive frequency distribution of the data was presented to check its normal distribution with subsequent outlier removals. The descriptive data were considered to understand the household energy use pattern and factors influencing households to use specific energy technologies. The study considered a cross‐tabulation of specific energy use against different household's characteristics to understand their patterns. Moreover, chi‐square test was performed to identify the association between categorical variables of interests, followed by logistic regression to determine the strength of their associations. Accordingly, the association of some household characteristics like family size, household's income, reliability of electricity connection, household's connection to grid and education of the wife with the households biomass energy use for cooking were tested with the logistic regression model. The statistical significance between the outcome variables and independent variables was determined at 95% confidence interval. Particulate emphasis was given to the factors determining the urban household's type of energy consumption for cooking and other services.

## 3 Results

### 3.1 Socioeconomic Characteristics of the Households

The socioeconomic characteristics of the households are presented in **Table** **1**. The finding shows that 34.3% of the respondents have a primary level education, and those with college and university‐level training accounts for 19.6%. The majority of the respondents were Orthodox Christianity followers (45.3%), followed by Muslim religion accounting for 38.1%. The income of the households is very diverse due to varying income sources of the households. The finding shows that 17% of the households reported that their monthly earning was less than 1000 ETB (29 USD), which is less than 1 USD a day, and 21.5% were making 1000–2000 ETB. Over 38% of the households were relying on less than 2 USD a day. Moreover, most households (61.1%) have a family size of 4–6 persons, and 3.8% have more than ten persons.

**Table 1:** Socioeconomic characteristics of the study population (*N* = 265)

	Category	Frequencies	[%]
Educational level	Primary school	91	34.3
	Secondary	39	14.7
	High school	83	31.3
	College and above	52	19.6
	Muslim	101	38.1
Religion	Orthodox	120	45.3
	Protestant	44	16.6
	Less than 1000	45	17.0
Household income in Eth birr (ETB)	1001–2000	57	21.5
	2001–4000	73	27.5
	4001–6000	37	14.0
	6001–8000	13	4.9
	8001–10000	23	8.7
	More than 10 000	17	6.4
	Less than 3	57	21.5
Family size	4–6	162	61.1
	7–9	36	13.6
	More than 10	10	3.8

### 3.2 Households Energy Characteristics with Different Energy Services

Household energy characteristics for cooking, lighting, and appliance services are presented in **Table** **2**. Households use different energy carriers, especially for cooking services, due to varying foods cooked at households. Households in Ethiopia mostly rely on “Injera” (a pancake‐like staple food prepared from cereal crops). Cooking Injera requires unique stoves and suitable energy types. Households use firewood, crops residues, sawdust, and electricity for cooking Injera, while charcoal and electricity are used for other cooking types like stew and coffee preparation. This is reflected in this study, where households use different energy carriers for cooking. Accordingly, 60% of the households use firewood, 98.1% use charcoal, and 69.4% use electricity for cooking. Over 90% of the firewood and 99% of the charcoal were obtained through purchasing (Figure 2a). The finding implies households’ dependencies on a dual‐energy system involving traditional and modern energy for cooking services; fuel stacking.

**Table 2:** Types of energy for different household energy services in Jimma town (*N* = 265)

Energy services	Energy type	Household responses	
		Yes [%]	No [%]
Cooking	Fire wood	159(60)	106(40)
	Agricultural residue	3 (1.1)	262 (98.9)
	Charcoal	260 (98.1)	5 (1.9)
	Grid electricity	184 (69.4)	81 (30.6)
	Sawdust	7 (2.6)	258 (97.4)
Lighting	Kerosene	25(9.4)	240(90.6)
	Candle	175 (66.0)	90 (34.0)
	Dry cell battery	56 (21.1)	206 (78.9)
	Solar PV	47 (17.7)	218 (82.3)
	Grid electricity	261 (98.5)	4 (1.5)
Appliances	Grid electricity	261(98.5)	4 (1.5)
	Solar PV	2 (0.8)	263 (99.2)

The lighting energy was not that much different from the cooking energy services concerning energy carriers. Households depend on multi‐energy sources despite their potential accessibility to grid connection. The majority of the households (98.5%) use grid electricity for lighting. Candle, solar PV, and kerosene were alternative energy sources for lighting used by 66%, 17.7%, and 9.4% of the households.

Similarly, those households (98.5%) using grid electricity for lighting also use it for appliances. Only less than 1% of the households use solar PV for the powering of devices. Households use energy for different types of appliances, including routine ones. Results in Figure 2b show that, the refrigerator was used by 54.3% of the households, Television by 83%, electric stoves by 69.4%, and over 95% use for rechargeable equipment such as mobile phones, hand battery, and rechargeable lamps.

This study further investigated the disaggregated biomass energy use for regular cooking by biomass energy types (**Table** **3**). Over 87% of the households use biomass for regular cooking services. Of all the households who regularly use biomass for cooking, 86.7% use charcoal, and 48.7% use firewood. This indicates that firewood and charcoal remain the households’ primary energy sources despite accessibility to grid electricity connections. Households use more than two energy sources for different cooking services. The results are to show the possibility of using each energy carrier by the households. Households may use firewood for cooking Injera, charcoal for cooking stew, and electricity for other meals at the same time.

**Table 3:** Household biomass energy for regular cooking services in Jimma town (*N* = 265)

Biomass use and its types	Household responses	
	Yes [%]	No [%]
Use biomass regularly	233 (87.9)	32(12.1)
• Firewood	129 (48.7)	136 (51.3)
• Crop residues	4 (1.5)	261 (98.5)
• Charcoal	230 (86.8)	35 (13.2)
• Sawdust	7 (2.6)	258 (97.4)

**Figure 1:**
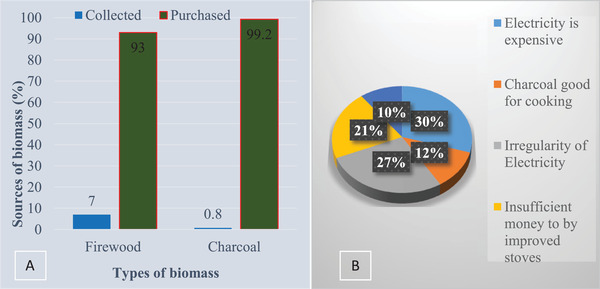
a) Types of biomass energy used by households, b) reason for regularly using biomass energy.

The amounts of biomass energy annually used by household cooking services are presented in **Table** **4**. Households mostly use firewood and charcoal for their cooking services. The amounts of each biomass type vary based on different factors. This can be attributed to cooking frequencies, family size, and types of food to be cooked. Households mostly cook Injera every two other days (two to three times per week), although other meals are cooked two to three times per day. Of those households using firewood regularly, 31.8% use less than 520 kg, and 26.4% use more than 1560 kg per year. The majority (64.8%) of the households use charcoal between 520 and 1040 kg per year for their regular cooking. Only 5.7% annually use more than 1560 kg—households on average use about 1236 kg of firewood and 630 kg of charcoal per year. Results in **Figure** **1**a show that 93% and 99.2% of the households purchase their firewood and charcoal.

**Table 4:** Yearly amounts of firewood and charcoal regularly used by households

Amount of biomass in kg	Number of households using each type of biomass	
	Firewood [%]	Charcoal [%]
<520	41 (31.8)	56 (24.3)
521–1040	37 (28.7)	149 (64.8)
1041–1560	17 (13.2)	12 (5.2)
1561–2080	18 (14)	9 (3.9)
>2080	16 (12.4)	4 (1.8)
Total	129 (100)	230 (100)

Households were asked to respond to why they are relying on biomass while having access to grid electricity. Accordingly, about 30% of them responded as electricity is expensive for cooking compared to firewood, and 27% of the households reported electricity power interruption as the main reason. Lack of insufficient money to buy improved stoves and preferences of charcoal for cooking were reported by 21% and 12%, respectively (Figure 1b).

Households were asked to respond to some practical questions related to their intention to use improved cooking energy technologies. Accordingly, 55.1% of the households replied as they do not have sufficient money to buy improved fuels and stoves (**Table** **5**). On the contrary, 90% of the households have reported a privately connected grid without sharing with their neighbors, which is mainly used for lighting and cooking Injera. About 68% of the households use biomass energy for cooking other foods, besides to use for cooking Injera. Moreover, 94% of the households complained about grid electricity connection and frequent power interruption. Households were also asked to respond to the energy types they regularly use to cook Injera and other food items (**Table** **6**). Accordingly, 55.8% and 40% of the households responded that they regularly use electricity and firewood for cooking Injera. The majority of the households (61.1%) regularly use charcoal and electricity (36.6%) for cooking other food items. This means that out of the 68.3% of the households using electricity for cooking other foods, about 50% of them use it for occasional cooking (Tables 5 and 6).

**Table 5:** Practical questions related to electricity use for cooking services (*N* = 265)

Questions	Responses	
	Yes [%]	No [%]
Do you have enough money to buy other fuels and modern stoves?	146 (55.1)	119 (44.9)
Do you have a private electricity connection?	238 (90.0)	27 (10.0)
Do you use electricity for cooking food other than injera?	181 (68.3)	84 (31.7)
Do you have a stable electricity connection?	16 (6)	249 (94)

**Table 6:** Households regular energy use for cooking Injera and other food items (*N* = 265)

Food types	Energy types	Frequency	[%]
Injera	Electricity	148	55.8
	Firewood	106	40.0
	Do not cook Injera	11	4.2
Other food items	Electricity	97	36.6
	Firewood	6	2.3
	Charcoal	162	61.1

**Figure 2:**
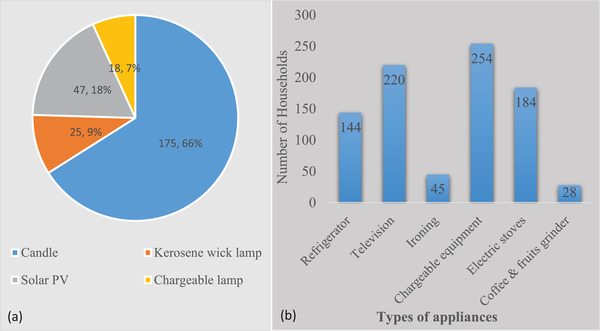
a) Households lighting energy when there is no electric connection. b) Types of appliances used by households in Jimma town.

As shown in Table 5, most households reported the irregularity of electricity connection and frequent blackout. During these times, households are forced to use other alternative energy sources for lighting. Accordingly, 175 (66%) of the households use candle, 47 (17.7%) use solar PV, 25 (9.4%) kerosene wick lamp, and 18 (6.8%) chargeable lamps (**Figure** **2**a).

The cross‐tabulation results disaggregated household monthly income and types of household energy consumption are given in **Table** **7**. As shown in the table, the likelihood of using electricity to cook and buy electric stoves proportionally increases with household income. Nevertheless, the use of biomass for regular cooking across the income category remains the same. The data clearly shows the stacking of biomass in the household energy system despite accessibility to electricity.

**Table 7:** Electricity from the interconnected grid for Cooking versus household income—a cross tabulation

Cooking energy services		Monthly household income in Birr							
		< = 1000	1001–2000	2001–4000	4001–6000	6001–8000	8001–10 000	>10 000	Total
Use electricity for cooking?	Yes	19	37	57	24	11	21	15	184
	No	26	20	16	13	2	2	2	81
Total		45	57	73	37	13	23	17	265
Use electric stoves?	Yes	21	40	52	23	12	19	17	184
	No	24	17	21	14	1	4	0	81
Total		45	57	73	37	13	23	17	265
Used biomass regularly?	Yes	43	53	61	31	10	20	15	233
	No	2	4	12	6	3	3	2	32
Total		45	57	73	37	13	23	17	265

Results on household's biomass energy use for cooking were tested with a binary logistic regression model to whether the household's strong dependency was determined by different household's characteristics. As shown in **Table** **8**, none of the parameters shown in the table became a single predictor of the present strong biomass use dependency. Household's level of education has shown statistically positive significant associations and households’ income have shown statistically negative associations. This implies that the level of awareness of the households and their income can affect the household's decision to use electricity for cooking rather than biomass. Having connection to electricity grid did not show statistically significant association with the use of biomass energy for cooking. This means that connection to grid electricity does not guarantee its use for cooking.

**Table 8:** Factors determining household biomass energy for cooking

Households’ characteristics					95% C.I. for EXP(*B*)	
	B	df	Sig.	Exp(*B*)	Lower	Upper
Household family size	‐0.243	1	0.180	0.784	0.550	1.119
Education of husband	0.319	1	0.011	1.376	1.077	1.758
Education of wife	0.414	1	0.000	1.514	1.210	1.894
Reliable electricity connection	‐0.164	1	0.752	0.849	0.306	2.353
Households’ income to afford modern stoves	‐0.554	1	0.031	0.575	0.348	0.950
Having connection to grid electricity	‐0.331	1	.232	.718	.417	1.236

## 4 Discussions

This study evaluated the typical urban household's energy profile for cooking, lighting, and appliances services. The results presented in Table 1 shows that households depend on different types of energy for the services. Ethiopian households cook different types of food. Injera is the main staple food cooked in most households. Cooking injera requires specific types of energy carriers depending on the kind of stoves used by households. Households use either electricity (if they use the electric stove); otherwise, firewood and sawdust are alternative energy. Households do not cook Injera every day; they cook only two to three times per week. Most households use electric and biomass stoves due to the probability of shifting when electricity is not available. In this survey, 60% of the households use firewood for cooking, mainly cooking Injera. The use of electricity for cooking Injera depends on the households’ economic capacity and connectivity to the grid. The data shown in Table 7 clearly shows the significance of household income to purchase electricity stoves and use for cooking. As household income increases, the likelihood of using electricity increases. Nevertheless, the data presented in Table 7 also show the stacking of the biomass energy in the household energy system remains serving as regular cooking services. The biomass stacking could be associated with the inefficient electricity system and frequent interruption, forcing households to rely on biomass energy.

Consequently, most poor urban households and those living in rented private houses may not have access to electricity for cooking. It is shown that about 80% of the surveyed households have private electricity connections, but 94% of them complained about its irregularity and blackout (Table 5). This could push households to remain reliant on biomass for cooking Injera. The result is consistent with the survey results reported in ref. [30]. Households use stew and different vegetables to eat with Injera. These food items are cooked at least one to two times a day, with relatively intensive energy use due to longer cooking hours. Households use different energy sources, including electricity, for cooking these food items; however, most households prefer charcoal. Coffee is also one of the traditional beverages in Ethiopia prepared two to three times a day, in which households mainly use charcoal to enjoy its ceremony. That could be why 98.1% of the households rely on charcoal for cooking (see Table 2).

As shown in Table 3, over 87% of the households use firewood and charcoal regularly for cooking despite their accessibility to grid connection. Such mixed‐use and household characteristics (i.e., family size, income, cooking behavior, etc.) could lead to varying amounts of biomass energy use among households. Of those households using firewood regularly, 37 (28.7%) of them annually use between 520 and 1040 kg compared to 149 (64.8%) of the households using the same amount of charcoal per year (Table 4). The variation between the two biomass types could be attributed to the frequency of use and the proportion of households relying on charcoal than firewood for most cooking. Households, on average, use about 1236 kg of firewood and 630 kg of charcoal per year. The finding is consistent with the literature results.^[^ ^31^, ^32^ ^]^ The annual difference between firewood and charcoal amounts could be attributed to their energy intensities and efficiencies. Charcoal provides an energy intensity twice that of firewood but requires 6–12 kg of wood to produce a kilogram of charcoal.^[^ ^31^, ^33^ ^]^ Moreover, different improved biomass stoves are available in urban areas to use charcoal than firewood for cooking.

The amount of firewood used in rural areas is high due to intensive use and combustion in traditional stoves; thus, much firewood is used compared to urban areas.^[^ ^34^ ^]^ However, there is a strong connection between rural and urban biomass energy sources. Over 99% of the charcoal and 93% of the firewood is purchased from rural areas (Figure 1a). Moreover, more than 99% of the rural regions rely on firewood for all cooking services.^[^ ^4^, ^30^ ^]^ This heavy reliance by rural and urban households has enormous environmental and economic implications.^[^ ^35^, ^36^ ^]^ Besides, the charcoal amount annually used by urban households (630 kg) requires 4000 –8000 kg of biomass. This indicates the intensive use of biomass energy by both rural and urban households for cooking. It is also essential to give due attention to the biomass energy business involved as income sources.^[^ ^31^, ^37^ ^]^ Unsustainable charcoal production inevitably leads to severe environmental consequences due to deforestation.^[^ ^38^, ^39^, ^40^ ^]^

The lighting energy is not intensive when compared to cooking fuel. Over 98% of households use electricity for lighting either from private or shared connections. Other energy sources, including kerosene wick lamp, could be an alternative when electricity is not available. Candle, Solar PV kits, chargeable lights, and batteries are the most dominant energy carriers used by households during power cuts. The result is consistent with the literature results.^[^ ^4^, ^28^ ^]^ Over 98% of the households connected to the grid use electricity to power appliances despite frequent power interruption. The majority (95.8%) of the households used electricity for charging equipment, 80% used for television, and 54.3% used for powering refrigerators (Figure 2b). The powering of refrigerators requires a continuous supply of electricity. As a result, households may not cook sufficient food at once and store it for later use. Hence, frequent cooking will significantly impact the amounts of energy used by households and related costs.

The provision of reliable, affordable, and sustainable energy supply is the goal to be achieved by Sustainable Development Goal 7 (SDG 7).^[^ ^23^ ^]^ Achieving goal 7 requires a complete transition from the traditional/inefficient energy system to the modern energy system. Investment in renewable energy and enhancing existing system efficiency is the strategy devised to progress towards the goal. The strategy could be plausible with respect to investment in renewable energy technologies because of the abundance of renewable energy sources in most countries deprived of modern energy services.^[^ ^41^, ^42^, ^43^ ^]^ Ethiopia is among countries deprived of access to modern energy technologies. The country is endowed with abundant renewable energy resources, mainly hydro, solar, and wind.^[^ ^4^ ^]^ Hydro and wind account for more than 90% of the country's energy supply, accessible to most urban areas.^[^ ^44^ ^]^ All urban households from the study area are potentially available to grid connection, and more than 80% already connected to the system (Table 5). However, most households use firewood and charcoal for cooking while having accessibility to grid connection. Only37% of the households reported a lack of private electricity connection and its irregularity as the main reasons for relying on biomass energy.

In contrast, large proportions of the households’ reasons were attributed to behavioral and attitudinal factors. This proves that connecting households to electricity does not guarantee the utilization of improved cooking energy technologies. Urban households’ fuel choice is determined by several factors associated with household income, education, cooking behavior, and characteristics; thus, perfect fuel switching cannot be possible.^[^ ^44^, ^45^ ^]^ Breaking cultural and behavioral factors would be challenging to enable all households to use modern energy technologies sustainably in the near future. Moreover, most developing countries depend on inefficient electricity systems involving a combined technical and nontechnical system loss of up to 50%.^[^ ^46^, ^47^ ^]^ Financial capacity is another major challenge to scale up the existing inefficient system and invest in new technologies to provide affordable and sustainable energy services.^[^ ^48^, ^49^, ^50^ ^]^ This implies that biomass energy remains as one of the main cooking energy supply in urban areas despite accessibility to grid connection. Energy policy and strategies aiming at urban energy transition should consider the existence of biomass energy in the supply system taking into account gradual shifting to electricity in the long run.

Achieving the goal (SDG 7) does not require only connecting households to electricity but also a system change. In system changing, we cannot tell what people cook and eat. Still, a system change focusing on technological modification based on sociocultural and economic contexts to meet local cooking energy needs. For instance, households in urban Ethiopia use electric stoves made of local materials to cook Injera. However, such kind of stoves is unaffordable to many of the poor households. Encouraging local innovators, producers, and beneficiaries in subsidies could enhance local technological adoption and penetration. Financial resources are essential to increase the accessibility and affordability of the technology to the poor being generated from different sources.^[^ ^49^ ^]^ Without comprehensive and well‐integrated action, increasing access to efficient and sustainable modern cooking energy technologies and achieving the goal is impossible. That means achieving the goal relies on the progress of other goals like SDG 1 aiming to end poverty for all people, SDG 4 focusing on quality education to encourage local innovation, and SDG 8 to ensure inclusive and sustainable economic growth. Moreover, achieving SDG 7 requires a better understanding of the local socio‐economic, cultural conditions, and available system infrastructure enabling system change.

## 5 Conclusion

This study evaluated the household energy profile of typical urban Ethiopia. The study area is one of the large towns where all households are potentially accessible to grid electricity. Accordingly, more than 80% of the households have a private grid connection, although they complained about the irregularity of the power and frequent interruption. However, more than 85% of households regularly rely on firewood and charcoal for basic cooking services. Most of the households’ reasons for not using electricity for cooking were mainly related to socioeconomic and behavioral factors as well as technical factors related to irregularity of electric energy supply.

Nevertheless, over 98% of the households use electricity for lighting, although the appliance's services are potentially limited to those having private electricity connections. Inaccessibility to modern cooking energy service cannot be solved simply by connecting households to electricity. Without a comprehensive and integrated policy focusing on holistic system change targeting socioeconomic development and behavioral change, providing modern energy for all households cannot be possible. Thus, biomass energy remains as one of the main cooking energy supply in urban areas despite accessibility to grid connection. Energy policy and strategies aiming at urban energy transition should consider the existence of biomass energy in the supply system taking into account gradual shifting to electricity in the long run. Without a comprehensive and locally focused policy achieving sustainable development goal seven and related goals cannot be realized. We recommend further study to have a full picture of suitable energy system to be considered in the policy reform.

## Conflict of Interest

The authors declare no conflict of interest.

## Data Availability

The data that support the findings of this study are available from the corresponding author upon reasonable request.
